# Anxiety and Depression among Patients with Chronic Kidney Disease Undergoing Haemodialysis in a Tertiary Care Centre: A Descriptive Cross-sectional Study

**DOI:** 10.31729/jnma.7608

**Published:** 2022-07-31

**Authors:** Rachana Sharma, Subhash Chandra Sharma, Pratikshya Chaiise, Jaya Regmee, Shaina Sharma

**Affiliations:** 1 Department of Psychiatry, Kathmandu Medical College and Teaching Hospital, Sinamangal, Kathmandu, Nepal; 2 Nobel College of Health and Educational Foundation, Sinamangal, Kathmandu, Nepal

**Keywords:** *anxiety*, *chronic kidney disease*, *depression*, *hemodialysis*

## Abstract

**Introduction:**

Chronic kidney disease is a global public health problem with psychological issues and other adverse issues like renal failure, cardiovascular disease, and premature deaths. This study aimed to find out the prevalence of anxiety and depression among patients with chronic kidney disease undergoing haemodialysis in a tertiary care centre.

**Methods:**

A descriptive cross-sectional study was done among patients with chronic kidney disease undergoing haemodialysis in the Department of Medicine, Nephrology unit of a tertiary care centre from December 2020 to June 2021. Ethical approval was taken from the Institutional Review Committee (Reference number: 1712202003). The whole sampling method was used. The diagnosis of anxiety and depression was made using the Nepali version of the Hospital Anxiety and Depression Scale with a cut-off of ≥8 scores.

**Results:**

Among 96 patients, the prevalence of anxiety was 66 (68.75%) and that of depression was 74 (77.08%) patients.

**Conclusions:**

The prevalence of anxiety and depression was similar to other studies done in similar settings.

## INTRODUCTION

Chronic Kidney Disease (CKD) is a global public health problem, with adverse outcomes of renal failure, cardiovascular disease, and even premature death.^1^ The patients with end-stage renal failure undergoing dialysis undergo major changes and adjustments in terms of their personal, professional, and social life.^2^ Once under dialysis, restriction of time and diet, unemployment due to illness, change in sexual interest and behaviour, increased financial burden and fear of death act as stressors, thus affecting the patient's mental health and quality of life.^2,3^

Depression and anxiety are the common mental health-related issues in patients undergoing haemodialysis due to CKD.^4^ Studies done in Nepal found the reduced quality of life and the prevalence of depression to be 75.5% and anxiety at 45.7% in patients with chronic kidney disease.^5^

This study aimed to find the prevalence of depression and anxiety among patients with chronic kidney disease undergoing haemodialysis in a tertiary care centre.

## METHODS

This descriptive cross-sectional study was conducted at the Department of Medicine, Nephrology unit of Kathmandu Medical College and Teaching Hospital after taking ethical approval from the Institutional Review Committee of the same institution (Reference number: 1712202003). The study was carried out from December 2020 to June 2021 among patients with chronic kidney disease undergoing haemodialysis. The patients aged 18 years and above, who provided written informed consent were included in the study. Whereas, the patients who were not in a clear sensorium while undergoing haemodialysis and were not able to provide written informed consent were excluded from the study. The whole sampling technique was used and all 96 patients during the study period were included in the study.

A semi-structured questionnaire was used to collect the socio-demographic details and other relevant information regarding haemodialysis. For the diagnosis of anxiety and depression, the Hospital Anxiety and Depression Scale (HADS) was used which is a self-rating scale first described in 1983 by Zigmond and Snaith.^6^ The scale contains two subscales which measured the symptoms of depression (HADS-D) and anxiety (HADS-A) present during the previous week. It includes seven statements on each disorder, and each response consists of a four-point rating scale (0 to 3); a higher score depicts a worse condition. For each subscale, the total score is at most 21 with a potential range from 0 to 21. For both anxiety and depression, a score of >11 is considered a clinically significant disorder, whereas a score between 8 and 10 suggests a mild disorder or borderline cases and a score between 0 and 7 signifies the normal range. The Nepali version of the HADS scale was used in our study for the diagnosis and a cutoff of >8 was used for both HADS-A and HADS-D.^7^ Consent for data collection was obtained from the patients and data regarding the diagnosis, sociodemographic details and details of the haemodialysis were collected.

The collected data were then entered and analysed using the IBM SPSS Statistics 20.0.

## RESULTS

Among 96 patients with chronic kidney disease who were undergoing haemodialysis, the prevalence of anxiety was 66 (68.75%) and the prevalence of depression was found to be 74 (77.08%). The mean age of the patients with anxiety was 51.06 ±16.38 years and the mean age of the patients with depression was 52.89±16.24 years.

Out of the patients with chronic kidney disease undergoing dialysis, 58 (60.40%) were male and 38 (39.60%) females. The mean age of participants was 52.40±15.52 years, the mean age of male participants was 50.93 ±16.75 and that of females was 54.40±13.40 years. A total of 93 (96.87%) patients had comorbid medical conditions, 51 (53.12%) had hypertension, 10 (10.41%) patients had diabetes mellitus and 32 (33.33%) patients had both diabetes mellitus and hypertension. A total of 50 (52.10%) patients had a history of smoking and 46 (51.11%) never smoked. None of the patients were smoking at present.

The mean HADS-A score among male patients with anxiety was 9.81±3.50 and among females with anxiety, the score was 10.18 ±3.81. Forty-one (62.12%) patients were males and 25 (37.87%) females. A total of 14 (21.21%) were Brahmins, 11 (16.67%) were Chhetris, 15 (22.72%) were Newars and 26 (39.39%) were from other castes. A total of 35 (53.03%) of the patients had a history of smoking and the mean year of smoking was 21.11±11.50 years.

However, the mean HADS-D score among male and female patients with depression were 10.00±3.80 and 10.05±4.21 respectively. A total of 44 (59.45%) patients were males and 30 (40.54%) were females. A total of 16 (21.62%) patients were Brahmins, 11 (14.86%) were Chhetris, 19 (25.67%) were Newars and the others were 28 (37.83%). A total of 38 (51.35%) of the patients had a history of smoking and the mean years of smoking were 21.15±11.17 years. The demographic details of the patients with anxiety and depression are tabulated below (Table 1).

**Table 1:** Demographic characteristics of the patients with anxiety and depression.

	Anxiety (n = 66) n (%)	Depression (n = 74) n (%)
**Marital Status**		
Married	56 (84.84)	64 (86.48)
Unmarried	10 (15.15)	10 (13.51)
**Educational status**		
Illiterate	22 (33.33)	26 (35.13)
Up to grade 10	27 (40.90)	31 (41.89)
Intermediate and bachelors	16 (24.24)	16 (21.62)
Masters and above	1 (1.51)	1 (1.35)
**Employment status**		
Employed	6 (9.09)	6 (8.10)
Unemployed	27 (40.90)	36 (48.64)
Unemployed after start of dialysis	33 (50)	32 (43.24)
**Monthly family income (Nepalese Rupee)**		
<25000	10 (15.15)	12 (16.21)
25000-50000	38 (57.57)	43 (58.10)
>50000	18 (27.27)	19 (25.67)

Thirty-four (51.51%) patients were hypertensive, 6 (9.09%) had diabetes mellitus and 24 (36.36%) had both hypertension and diabetes mellitus among patients with anxiety. Similarly, 38 (51.35%) patients were hypertensive, 12 (16.21%) had diabetes mellitus and 28 (37.83%) had both hypertension and diabetes mellitus among patients with depression. The haemodialysis details of these patients are tabulated below (Table 2).

**Table 2:** Dialysis details of the patients with anxiety and depression.

Characteristics	Anxiety (n= 66) n (%)	Depression (n= 74) n (%)
**Duration of dialysis (years)**		
<1	34 (51.51)	37 (50)
1 to 5	15 (22.72)	36 (48.64)
>5	17 (25.75)	19 (25.67)
**Frequency of haemodialysis per week**		
Once a week	-	1 (1.35)
Twice a week	66 (100)	73 (98.64)

Mean expenditure per cycle of dialysis is 2736.36±398.70 NPR for patients with anxiety and 2727.02±402.83 NPR among patients with depression.

## DISCUSSION

Our study showed that the prevalence of depression and anxiety among patients undergoing haemodialysis due to chronic kidney disease was 77.08% and 68.75% respectively. Our finding is similar to a multicentric study done in Nepal which showed the prevalence of depression to be 75.50% in patients with chronic kidney disease.^5^ Another hospital-based study conducted among patients undergoing haemodialysis due to chronic kidney disease showed the prevalence to be 78% which is similar to our study.^8^ A study conducted in Rajasthan, India reported the prevalence of depression and anxiety to be 66% and 61% respectively which is quite similar to our study.^9^

One study conducted in Nepal found the prevalence of depression to be 51.8% which is lower than our study.^10^ A study by Maharashtra, India, concluded that depression was the most common psychiatric comorbidity in patients undergoing haemodialysis (40.69%)." Another study conducted in the Middle East found the prevalence of depression and anxiety among patients undergoing haemodialysis to be 56.4% and 51.3% respectively.^12^ A study conducted in Brazil showed that the symptoms of depression and anxiety were present in 41.70% and 32.30% respectively of those undergoing dialysis.^13^ The prevalence of anxiety and depression is seen to vary among different studies conducted mainly because of the difference in study design, diagnostic criteria used, study population, and tools used in the study but in general, it can be said that the occurrence of depression and anxiety is high in a patient with chronic kidney disease undergoing dialysis.

CKD is a long-standing medical illness, the chronicity associated with it is usually perceived as significant stress. The chronicity of illness means limitation and restrictions in everyday life, readjusting, and coping to the perception of self (loss of bodily function, insecurity about job, limitation of physical activity etc.), families, potential changes in the relationship with spouse and even community. All of these can affect the mental health of an individual predisposing them to psychiatric comorbidities like depression and anxiety.^2,3,14^ The progressive nature of this disease, along with the medical conditions like anaemia, uremia etc. and the associated psychological factors like uncertainty about health, treatment options and outcomes, constant worry about finances, and chances of medical crisis during illness and treatment may all predispose to the occurrence of anxiety.^9,12^

There is inconsistency in the available literature regarding the association of the gender of the patients with anxiety and depression. Some studies have shown a strong correlation between these variables whereas such correlation is absent in other studies.^14,15^ The prevalence of both anxiety and depression in our study was found to be higher among males when compared to females in our study. Further, in our study, we observed depression and anxiety to be more common among patients who were undergoing dialysis for less than a year. One study done in Nepal reported similar findings where the rate of depression was higher among those undergoing dialysis for less than one year.^10^ One possible reason for this finding may be that being diagnosed with chronic illness leading to significant changes in one's lifestyle may acutely lead to the development of psychological disorders, which with time patient may become adjusted to the situation.

There were some limitations in this study. As this was a descriptive cross-sectional study, only the prevalence of the conditions could be studied and association or correlation among the study variables could not be made. Also, causality could not be established in our study. Further, the small sample size and single-centric nature of the study limit the generalizability of the findings of the study.

## CONCLUSIONS

The study concluded that the prevalence of depression and anxiety among patients with chronic kidney disease undergoing haemodialysis was similar to other studies done in similar settings. The finding highlights the importance of early psychiatric evaluation and intervention in this group of people.
